# The Dangers of Water Gas

**Published:** 1885-04

**Authors:** 


### THE DANGERS OF WATER-GAS.

The Boston *Medical and Surgical Journal* for March 5th contains an editorial on this subject that is timely. It is a matter of so much importance to our people who are using this dangerous gas that we reproduce it, entire, and commend it to their careful consideration:

Considered as a sanitary matter, the manufacture and use of water-gas for domestic purposes cannot be defended for a moment. On the contrary, every consideration of safety is opposed to the introduction of this dangerous agent into dwelling houses and public buildings. The source of the danger lies in the relatively great amount of carbonic oxide which is left in the product of the process of converting steam into gas by its exposure to incandescent anthracite. As is well known, carbonic oxide is of all gaseous poisons the most lethal; it kills as surely, if not as quickly, as hydrocyanic acid, and to admit it to inhabited rooms as an illuminating agent, under even more than ordinary safeguards against leakage, is an experiment the risk of which can not be denied.

These are trite sayings. Yet the energetic agents of speculative enterprises, eager to place their "plant" and to make money out of the dear public, will declare that there is no danger in water-gas worth mentioning. They catch the attention by emphasizing the economic side of the case, and by reiterating how cheaply they can supply the new article. They wish all statutory restrictions removed so that, in the name of economy, they may make and send through our streets and into our houses an illuminating gas containing thirty per cent, more or less, of carbonic oxide. They grow hilarious when their attention is called to the record of deaths by the inhalation of the "improved" gas in cities which have surrendered to their persuasive overtures. They declare that the coal-gas ordinarily used for illuminating purposes contains carbonic oxide like their water-gas, but that no one thinks of going back to whale oil or tallow candles through any apprehensions on that account. In short, they sneer at all the cautions which unprejudiced and unpurchased sanitary chemists have uttered since the composition of water-gas was first appreciated, and they, in effect, ask the people to take a catamount or a lioness into their laps to caress in place of the familiar domesticated animal which gives its name to the whole feline family. Sensible persons will hesitate before they try the experiment, and will choose rather to endure the gas they have than to fly to other gas four or five times more dangerous to life.

These reflections are suggested by a report of recent investigations made under the instructions and direction of the Massachusetts Board of Health, Lunacy, and Charity, by Professors Sedgwick and Nichols, of the Institute of Technology, in accordance with an order of the Massachusetts Legislature. The report, though only preliminary to a fuller exposition of the subject after further experiments have been made by the authors, is very convincing. After a general statement of the nature and composition of illuminating gas, of which "the only ingredient possessed of really toxic properties is carbonic oxide," this being "intensely poisonous," the report presents a summary of the conclusions to which the authors have been led by their experiments. Of these we give a condensed abstract:

(1) Water-gas is decidedly more poisonous than coal-gas.

(2) An atmosphere containing a small percentage of coal-gas may be breathed many hours without serious effects, while an atmosphere containing the same amount of water-gas will be injurious and even fatal.

(3) On account of natural ventilation constantly going on in the rooms, thus permitting considerable diffusion, ordinary coal-gas, containing about seven per cent of carbonic oxide, is not a source of serious danger; with water-gas, on the other hand, on account of the larger proportion (thirty per cent) of carbonic oxide, the danger line is easy to reach. And it must not be inferred that a gas containing twice as much carbonic oxide as another is necessarily only twice as dangerous. Water-gas is not only in itself more poisonous than coal-gas, but is also far more likely to produce injurious effects from similar accidental causes.

(4) Dogs, cats, rabbits, and pigeons did not show any symptoms of poisoning after exposure for many hours to an atmosphere containing one per cent of coal-gas, being apparently able to resist it almost indefinitely; but the same animals and birds when exposed, under the same conditions, to an atmosphere containing from one-half to one per cent of the water-gas invariably showed marked symptoms of poisoning at the end of an hour and a half, and death generally resulted after from five to eight hours of exposure to an atmosphere containing not more than one per cent.

(5) If instead of comparing the effects of the same percentage of the two gases, we consider the *time* necessary to cause poisoning by the use of the same quantities of gas under the same conditions, we find a contrast not less striking. With water-gas let into a closed chamber of known capacity (700 cubic feet) at the rate of six feet per hour, the animals under observation showed well-developed symptoms of poisoning in an hour and a half, and were all dead within eight hours. In a corresponding experiment with coal-gas, a similar set of animals presented symptoms from which recovery would have been possible, and even easy, had they been set free; after twenty-four hours of continuous exposure, one cat and one rabbit were dead, but the other animals (dogs, cats and rabbits,) were not even unconscious.

It cannot excite surprise that, after these results, the report should contain these words in its closing paragraph: "Our opinion, based upon experiments, is decidedly averse to the general distribution of the so-called water-gas." Earnestly solicitous to promote all measures for public health and to oppose the advance, insidious or open, of all projects having a contrary tendency, we commend these independent and trustworthy observations of Professors Sedgwick and Nichols to the attention of our legislatures, and hope none of them will be tempted by false doctrines or other considerations to rescind the wise provision which forbids the manufacture of gas containing more than ten per cent of carbonic oxide.

